# Yoga Plus Mantram Repetition to Reduce Chronic Pain in Veterans With Post-Traumatic Stress Disorder: A Feasibility Trial

**DOI:** 10.1177/27536130231220623

**Published:** 2023-12-26

**Authors:** Erik J. Groessl, Carol Hafey, Adhana McCarthy, Rahil M. Hernandez, Miguel Prado-Nava, Danielle Casteel, Symone McKinnon, Douglas G. Chang, Catherine R. Ayers, Thomas R. Rutledge, Ariel J. Lang, Jill E. Bormann

**Affiliations:** 1 19979 VA San Diego Healthcare System, San Diego, CA, USA; 2 Herbert Wertheim School of Public Health, 8784 University of California San Diego, La Jolla, CA, USA; 3 UCSD Health Services Research Center, San Diego, CA, USA; 4 US Army, San Antonio, TX, USA; 5 Physical Medicine and Rehabilitation, Department of Orthopedic Surgery, 8784 University of California San Diego, La Jolla, CA, USA; 6 Department of Psychiatry, 8784 University of California San Diego, La Jolla, CA, USA; 7 Hahn School of Nursing and Health Sciences, Beyster Institute of Nursing Research, University of San Diego, San Diego, CA, USA

**Keywords:** veterans, chronic pain, post-traumatic stress disorder, yoga, mantram repetition

## Abstract

**Background:**

Veterans with post-traumatic stress disorder (PTSD) are more likely to report chronic pain than veterans without PTSD. Yoga has been shown to reduce both chronic pain and PTSD symptoms in clinical trials. The goal of our study was to assess the feasibility and acceptability of conducting a randomized controlled trial (RCT) that combined yoga and mantram repetition (Yoga + MR) into one program for military veterans with both chronic pain and PTSD.

**Methods:**

In this feasibility RCT, 27 veterans were randomized to either Yoga + MR or a relaxation intervention. Due to the COVID-19 pandemic, in-person recruitment, assessments, and intervention attendance were re-evaluated. Although remote delivery of aspects of the study were utilized, interventions were delivered in-person. Feasibility benchmarks met included full recruitment in 12 months or less, 75%+ retention at initial follow-up assessment, 50%+ attendance rate, and 75%+ of participants satisfied with the interventions.

**Results:**

The sample was racially and ethnically diverse, and 15% of participants were women. Participant recruitment lasted approximately 11 months. Out of 32 participants initially randomized, two participants asked to be dropped from the study and three did not meet PTSD symptom criteria. For the remaining 27 participants, retention rates were 85% at 12 weeks and 81% at 18 weeks. Participants attended 66% of in-person yoga and 55% of in-person relaxation sessions. Satisfaction was high, with 100% of yoga participants and 75%/88% of relaxation participants agreeing or strongly agreeing they were satisfied with the intervention/instructors. After 12 weeks (end of intervention), Yoga + MR participants reported reduced back-pain related disability (primary outcome), reduced alcohol use, reduced fatigue, and increased quality of life, while relaxation group participants reported reductions in pain severity, PTSD symptoms, and fatigue.

**Conclusions:**

Amidst many research challenges during the pandemic, recruitment, retention, and efficacy results from this feasibility trial support advancement to a larger RCT to study Yoga + MR for chronic pain and PTSD.

## Introduction

Musculoskeletal disorders are the most common type of chronic pain conditions reported by Veterans.^1,2^ Within musculoskeletal conditions, chronic low back pain (cLBP) and chronic neck pain (cNP) are two of the most common reasons for disability among deployed military personnel^3^ and the military in general.^4^ Although opioid medications were previously a front-line option for treating chronic pain,^5,6^ the higher risk of addiction in veteran populations and subsequent adverse outcomes including overdose deaths^7^ have led to increased efforts to provide non-pharmacological pain treatments,^8^ and reduce long-term opioid therapies in the VA system.^9,10^ Current guidelines now recommend non-pharmacological treatments as a first-line option for all people with cLBP.^10,11^

Post-traumatic stress disorder (PTSD) afflicts thousands of Veterans and is associated with a broad array of physical and functional impairments in addition to PTSD-specific symptoms.^12,13^ Recent research indicates that Veterans with PTSD are also more likely to report the presence of chronic pain,^14^ and they report greater pain-related disability and pain severity than those without PTSD.^15^ Although the exact reasons are still unclear, Veterans with both chronic pain and PTSD have proven difficult to treat,^16^ possibly because of other common co-occurring factors such as substance use^17,18^ and/or mild traumatic brain injury (mTBI).^19,20^

Mind-body interventions are a subset of the non-pharmacological treatment options that have a growing evidence base and comparatively few side effects for treating persons with pain or PTSD.^21,22^ Among mind-body therapies, yoga has a strong evidence base for reducing cLBP-related disability and pain severity in both Veterans^23^ and non-Veterans.^21,24,25^ As a result, yoga has been included in recent reviews and clinical practice guidelines as a recommended treatment option.^21^ Yoga has also been found to be beneficial for persons with chronic neck pain (cNP).^26^

A growing body of evidence also suggests that yoga can be effective for treating PTSD,^27^ with a recent RCT finding significant benefits of yoga for reducing symptoms and restoring function among Veterans with PTSD.^28^ Other mind-body interventions such as mantram repetition (MR)^29-31^ and Mindfulness-Based Stress Reduction (MBSR)^32,33^ are also supported as effective treatment options for Veterans with PTSD.

Thus, given that chronic pain and PTSD are commonly co-occurring, treatment-resistant conditions that have been shown to respond to mind-body interventions, we sought to expand an existing Yoga for cLBP program to military veterans with both chronic pain and PTSD. Some prior research points to the potential of treating pain and PTSD, as co-occurring conditions, with integrated non-pharmacological interventions.^34-36^ One recently published study of yoga for treating Veterans with both chronic pain and PTSD found improvements, but the study did not have a control group, had rather high attrition rates, and did not include a mantram repetition (MR) component.^37^

The rationale for integrating MR with yoga was, first, that MR has been shown to improve PTSD symptoms^31,38^ and research indicates that pain and pain-related disability is significantly harder to treat when PTSD is co-occurring.^22^ Second, when practiced regularly, MR provides a portable tool for calming and centering oneself in almost any environment which may facilitate the reintegration of Veterans into civilian life; a main goal of VA research programs.^39^ Yoga asanas (poses) are not easily practiced in some public settings, and deep breathing may draw attention to oneself or may exacerbate anxiety in some individuals.^40^ MR is easily practiced in almost any setting since it involves silently repeating a spiritual or meaningful phrase to oneself.^30^ Although attention to one’s breath can be used to promote calmness without drawing attention to oneself, MR also adds the benefit of a personal, spiritually meaningful phrase and associated cognition.^30^ When linked to a yoga practice, Yoga + MR could strengthen resiliency and possibly reduce both pain and PTSD symptoms.

The purpose of this pilot study was to combine the existing yoga and MR programs into a single program and to test the feasibility and acceptability of conducting a randomized controlled trial of Yoga + MR compared to a relaxation comparison group for veterans with chronic pain and PTSD.

## Methods

### Study Design

The study was a pilot randomized controlled trial to examine the feasibility of recruitment, retention, assessments, and randomization, along with the safety and acceptability of an enhanced Yoga + MR intervention for reducing disability and chronic pain among Veterans with PTSD. Eligible patients at a large VA Medical Center with either cLBP and/or cNP who experience clinically significant symptoms of PTSD (n = 32 total) were randomized to either Yoga + MR or a relaxation control intervention. Participants in both intervention groups attended class once a week for 12 weeks and completed home-based activities between weekly sessions. The Yoga + MR group sessions consisted of about 15 minutes of instruction on MR followed by an hour of yoga. The relaxation intervention was 75 minutes of classroom instruction and group discussion. Both Yoga + MR and the relaxation classes were conducted in person at VA medical facilities with adherence to COVID-19 pandemic-related safety protocols. Participant recruitment occurred from October 2020 to September 2021. Interventions and assessments occurred between April 2021 and February 2022. More detailed information on the interventions used in this study is available from the corresponding author.

The primary purpose of the study was to measure feasibility in terms of recruitment rates, intervention attendance rates, safety, and satisfaction. Health outcomes were assessed through questionnaires and physical performance tasks at baseline, 12 weeks, and 18 weeks. The 18-week assessment examined the feasibility of retention at 1 point after the intervention had concluded. The target sample size was 32 participants recruited via 2 cohorts of roughly 16 participants each, resulting in about 8 participants assigned to each intervention group in each cohort. The study protocol was reviewed and approved by the VA San Diego (VASD) Institutional Review Board (IRB) and Human Research Protection Program (HRPP).

### Recruitment

The primary method of recruitment was through health care provider referral. The study was described and promoted by study staff to VASD health care providers (mental health, primary care, pain medicine, physical therapy) at regularly scheduled online clinical team meetings. Participants were also recruited through posted flyers. Flyers were displayed in primary care, mental health, and pain clinics as well as given to physicians in these clinics to distribute. Advertisements were also sent to virtual message boards in the VASD and surrounding VA clinics. Recruitment options were limited by research restrictions from the COVID-19 pandemic, which prohibited in-person recruitment or in-person study presentations to clinicians.

### Inclusion/Exclusion Criteria, Consent, and Screening

Eligible participants were US military veterans who were VASD patients with a clinical diagnosis of cLBP and/or cNP (low back and/or neck pain greater than 3 months duration) and symptoms of PTSD (PCL-5 of ≥20 or a medically documented diagnosis of PTSD). A lower PCL-5 cutoff was used to be inclusive considering fluctuation of symptoms. Willingness to attend 12 weeks of a mind-body intervention and complete three assessments was also required. Exclusion criteria were serious or unstable psychiatric illness (eg psychosis, mania), active suicidal or homicidal intent (C-SSRS endorsed items 4 or 5), <3 months since major trauma event, moderate or severe cognitive impairment (MoCA score <18), practiced yoga > twice in the last 6 months, or coexisting medical illness with yoga contraindicated.

Potential participants inquiring about the study underwent a pre-screen by phone in which study criteria and procedures were explained but personal health information was not requested or recorded. The interventions were described as two different types of mind-body interventions for improving pain and PTSD symptoms and no indication was given that one intervention might be more effective than the other. If the potential participant remained interested and believed they would qualify, they were scheduled for a formal in-person screening appointment. Screening appointments consisted of three components. First, informed consent and permission to access medical records were provided for the study. This was followed by a questionnaire-based assessment by research staff and a clinical examination plus medical record review conducted by a clinician. Research staff administered the Montreal Cognitive Assessment (MoCA), the Mini International Neuropsychiatric Interview (MINI),^27^ and, when indicated, the Columbia Suicide Severity Rating Scale (C-SSRS). The C-SSRS was used when suicidal ideation was present and/or the MINI suicidality score suggested a moderate to high suicide risk. If the C-SSRS indicated active suicidal intent or plan, the assessment was stopped and the patient was referred to the VASD Suicide Prevention Team or VASD Psychiatric Emergency Clinic. The second part of the screening assessment was conducted by licensed VA clinicians (the study physician or a physician assistant). Prior to the appointment, the clinician reviewed the electronic medical record (EMR) and then conducted a clinical interview focused on chronic low back or neck pain followed by a brief physical examination to apply study criteria and promote safe participation.

Eligible participants completed the baseline assessment and were randomized. Randomization was implemented by the project coordinator using a computer program (1:1 ratio, 1 block of 32 to balance groups) created by a statistician. Allocation was concealed and only available upon randomization. Screening for study criteria, baseline assessments, and randomization were combined into a single appointment for the convenience of participants, which was especially important during the pandemic. However, this resulted in the preliminary enrollment of three participants who were later determined to be ineligible. They were initially enrolled because of medical record evidence of a PTSD diagnosis. It was later determined they had minimal PTSD symptoms and the diagnoses were outdated.

### Retention and Attendance

During the baseline appointment, participants completed questions to assess barriers to attendance and participation. This information was reviewed by research staff and the importance of attendance and completing all assessments was discussed with participants. Retention efforts included phone calls and emails to schedule and provide reminders for assessments and intervention sessions. Research staff attempted to contact all participants who missed intervention sessions to assess for adverse events and encourage future attendance. When participants were unable or unwilling to attend assessments, one or more phone appointments for assessments were made to accommodate them. Participants were compensated $30 for the completion of each assessment, and they were given $5 per intervention session attended to offset transportation costs. Each intervention was offered on one weekday and one weeknight to facilitate attendance.

### Yoga + MR Intervention

The existing evidence-based yoga intervention^23,41^ was adapted by therapeutically oriented yoga instructors with over 10 years of experience to benefit Veterans with cNP and to be more trauma-informed.^40^ Participants were asked to attend yoga + MR sessions once weekly for 12 weeks. Participants received a yoga home practice manual containing basic, safe postures that could be performed in about 20 minutes. The yoga intervention was classic hatha yoga with Iyengar influences. It uses multiple pose modifications and props to make poses accessible to those limited by health problems and to minimize injury risk.^42^ The intervention included 25 yoga poses, some with multiple variations, performed at a slow to moderate pace. Yoga lasted 45 minutes in Week 1 and about 14 poses were available. In weeks 2-12, up to 20 poses were offered over 60 minutes. However, participants varied in their ability and some participants did slightly more than others. For variability in the regimen, the instructor manual included 2 different sequences that alternated every 2 weeks. Participants were instructed to take slow, deep breaths, be intentional in their practice, focus their attention in conjunction with poses and movement, and to emulate the postural alignment modeled by the instructor. Sessions began with breathing and meditation followed by 10-15 minutes of basic postures to warm-up, increase circulation, and increase flexibility. This was followed by a series of standing poses for about 15-20 minutes. Next, the class transitioned to floor poses for about 15-20 minutes. Each session ended with about 5-10 minutes of relaxation in supine resting pose or “savasana”. Trauma-sensitive adaptations included the use of invitational (as opposed to commanding) and calming language, allowing patients to opt out of postures, and minimizing physical assistance while trust was established.^40^

The Mantram Repetition Program (MRP) is a portable meditative intervention that has been shown to be effective for improving PTSD symptoms.^31,38^ MRP teaches portable tools including (a) repeating a self-selected “mantram”: a word or phrase that provides spiritual strength or meaning for that individual; (b) slowing down thoughts and increasing awareness; also setting priorities to avoid feeling rushed; and (c) one-pointed attention or concentrating on one thing at a time, thereby staying in the present moment and conserving energy. The MRP was condensed and manualized to be taught in conjunction with yoga over 12 weeks. Week 1 included 30 minutes of MR training, followed by 45 minutes of yoga. Weeks 2 through 12 included about 15 minutes of MR training and dialogue, followed by 60 minutes of yoga. Participants were strongly encouraged to practice MR between weekly sessions during daily life activities and were asked to self-monitor their use of MR with weekly logs.

The Yoga + MR intervention was led by two different instructors, with each instructor teaching both Yoga and MR at different times during the week. They each had 15-20 years of yoga teaching experience including prior trauma-sensitive yoga training. Both instructors collaborated with the study investigators on the adaptation of the intervention from prior programs and thus were extensively familiar with the intervention as designed. The instructors also underwent 12 hours of MR training. These collaborative trainings were meant to promote intervention fidelity. However, because the combined intervention was novel, elements were compressed into shorter timeframes, and there were numerous challenges due to the pandemic (i.e. face masks required during yoga), the study investigators did not emphasize or formally measure fidelity. Although based on established interventions, it was unclear whether the interventions (mostly MR) could be properly delivered in the time allotted. Instead, fidelity was informally studied through weekly discussions designed to understand intervention delivery timing, the impact of COVID restrictions, and any aspects of the intervention that needed to be altered.

### Relaxation Intervention

The comparison intervention was a PTSD health education intervention that focused on relaxation techniques. The intervention was adapted from the Veteran Calm (VC) “mind-body intervention” used previously.^43^ VC was derived from work by Taylor et al.^44^ When chosen, this intervention was viewed as an active control intervention that was unlikely to significantly impact chronic pain or PTSD based on minimal effects in the prior study.^43^ The 12 weekly 75-minute sessions featured psychoeducation about PTSD and the rationale for relaxation therapy, along with relaxation techniques (progressive muscle relaxation, imagery), sleep hygiene, applied relaxation, and in-session relaxing experiences (self-massage, chair stretching, sensory experiences). Participants were given handouts and encouraged to practice relaxation exercises at home. Sessions were led by an experienced health educator.

## Measures

Feasibility, acceptability, and safety were measured by recruitment rates, attendance rates, retention at follow-up assessments, satisfaction with the intervention and instructors, and adverse event (AE) data. Information on the timeframe and referral source were maintained during recruitment, and all aspects of study participation were documented by research staff. Attendance was tracked with a sign-in sheet that included a question about the amount of home practice performed during the week. Satisfaction was measured by questions using scaled and free text response options, given at the 12-week follow-up assessment. An open-ended question allowed for additional comments. AE information was collected in class via a weekly log. Participants were instructed to contact intervention instructors or study staff if they experienced any health problems possibly related to the interventions. AEs were assessed by phone if intervention sessions were missed.

A priori feasibility cutoffs were proposed as follows: A recruitment rate of 4 enrolled participants per month. Recruitment rates below 50% of the goal would suggest that feasibility was not established in preparation for a full-scale RCT. Attendance rates in many mind-body intervention studies range between 50%–80%.^45-47^ For this study, attendance of 50% or higher was targeted to support further research. A retention rate of 80% or higher is considered an adequate retention rate at a post-intervention assessment for an RCT.^48^ For this study, a slightly more lenient rate of 75% or higher retention was set to indicate feasibility. Participant satisfaction rates of 75% or higher (% of participants that agree or strongly agree with the satisfaction statement) indicated intervention acceptability. For AEs, any serious AEs directly attributed to either intervention would indicate a need for reconsidering the intervention before a larger study.

Health outcomes were assessed at baseline, 12-week, and 18-week consisting of self-report questionnaires. Participant demographic information was assessed with a brief questionnaire. The VA medical record was used to verify participant medical histories and co-morbid disorders. Staff conducting data collection, data entry, and data management were blinded to group assignment. Pain-related disability was chosen as the primary outcome because functional restoration for veterans served by the VA healthcare system is a major part of the mission of VA Rehabilitation Research and Development.^39^ Additionally, functional limitations or interference with function has often been viewed as more important to those with chronic pain than actual pain severity,^49^ which may be more subjective. Pain-related disability has been used previously in many other studies of chronic pain.^23,50^

Back pain-related disability was measured using the 24-question Roland-Morris Disability Questionnaire (RMDQ). The RMDQ is a valid and reliable way^30^ to measure level of disability and is sensitive to change over time. Pain severity and interference were measured with the Brief Pain Inventory (BPI) with 4 questions related to severity and 7 questions related to interference.^31^ Symptoms of PTSD were measured using the Post-Traumatic Stress Checklist (PCL-5), a self-report tool measuring symptoms of PTSD according to DSM-5.^32^ Insomnia was measured using the Insomnia Severity Index (ISI).^34^ Alcohol use was measured in terms of typical use and binge drinking using the Alcohol Use Disorders Identification Test-Concise (AUDIT-C).^33^ One modification was to allow participants to choose zero in response to the question “How many standard drinks containing alcohol do you have on a typical day?” to account for participants who do not drink. Health-related quality of life was measured using the EQ5D-5L.^51^ Fatigue was measured using the Fatigue Severity Scale (FSS).^52^ Depression was measured using the Patient Health Questionnaire (PHQ-9).^53^

### Medication and Other Pain Treatments

Medication data were abstracted from the VA electronic medical record (EMR). Opioid medication taken during participation in the intervention was recorded. Other pain medications recorded included ibuprofen, 5% lidocaine patch, acetaminophen, naproxen, meloxicam, and a field for “other” medications. The use of antidepressants or other medication sometimes prescribed to help treat chronic pain were also recorded.^35^ Other non-medication pain treatments used at home or outside of the VA were assessed using self-report questionnaires.

### Statistical Analysis

As a feasibility study, the sample size was designed to provide information on feasibility metrics. The sample size and study design were not designed or powered to evaluate statistically significant differences between groups or within groups over time for health outcomes (disability, pain, PTSD symptoms, etc.). Guidelines for conducting and reporting the results of feasibility trials advocate against presenting *P*-values for health effects.^54^ Thus, we provide pre-post effect sizes and 95% confidence intervals for the pre-post change in outcomes. Means and raw data were reported for measures of feasibility. Although we are not conducting significance testing for measures of feasibility or health outcomes, we report means and thus examined variable distributions and tested each for normality. T-tests and chi-square analysis were used to examine the magnitude of baseline differences.

## Results

The final enrolled sample (n = 29) included 4 women (14%) and 25 men ranging in age from 23 to 79. Participants self-reported that they were 45% White, 14% Black or African American, 17% more than 1 race, 7% Native Hawaiian or other Pacific Islander, and 3% American Indian or Alaska Native, with 14% not responding. Thirty-eight% of participants identified as Hispanic or Latino. Detailed characteristics of the sample are shown in Table 1. Relaxation group participants were significantly younger than Yoga + MR participants (40.1 vs 51.9 years respectively; *P* = .024) and had a higher proportion of women (X^2^ = 5.01; *P* = .025). Other baseline differences were not statistically significant.

**Table 1.** Demographics.

	Yoga + MR (n = 14)	Relaxation (n = 15)
Age* mean (*SD*)	51.9 (13.5)	40.1 (10.7)
Gender*		
Male	14 (100%)	11 (73%)
Female	0 (0%)	4 (27%)
Other	0 (0%)	0 (0%)
Military branch		
Navy	7 (50%)	7 (47%)
Marines	5 (36%)	3 (20%)
Air force	0 (0%)	0 (0%)
Army	1 (7%)	5 (33%)
National guard	1 (7%)	0 (0%)
Combat service		
Yes	7 (50%)	11 (73%)
No	7 (50%)	4 (27%)
Ethnicity		
Hispanic/Latino	6 (43%)	5 (33%)
Not hispanic/Latino	8 (57%)	10 (67%)
Race		
White	7 (50%)	6 (40%)
Black or African American	2 (14%)	2 (13%)
Asian	0 (.0%)	0 (.0%)
American Indian or Alaskan native	0 (.0%)	1 (7%)
Native Hawaiian or other Pacific Islander	1 (7%)	1 (7%)
More than 1 race	2 (14%)	3 (20%)
Not available	2 (14%)	2 (13%)
Marital status		
Single-never married	1 (7%)	3 (20%)
Cohabitating	1 (7%)	0 (0%)
Married	6 (43%)	10 (67%)
Divorced or separated	4 (29%)	2 (13%)
Widowed	2 (14%)	0 (.0%)
Employment		
Disabled or unemployed	3 (21%)	7 (46%)
Full-time	4 (29%)	3 (20%)
Retired	6 (43%)	1 (7%)
Did not answer	1 (7%)	4 (27%)
Education		
High school or equivalent	0 (0%)	5 (33%)
Technical school or college	0 (0%)	1 (7%)
One or more years of college	5 (36%)	4 (27%)
Associate degree	3 (21%)	2 (13%)
Bachelor’s degree	4 (29%)	2 (13%)
Graduate degree	2 (14%)	1 (7%)
Household income		
$10K – $39K	3 (21%)	3 (20%)
$40K – $100K	7 (50%)	9 (60%)
$100K or more	4 (29%)	3 (20%)
Clinical characteristics – mean (SD)		
RMDQ score	13.3 (4.6)	14.8 (6.5)
BPI Pain severity	4.8 (1.8)	5.6 (1.9)
BPI Pain interference	5.3 (2.4)	6.0 (2.4)
PCL-5 score	40.8 (14.6)	59.5 (15.4)
Pain duration – mean (SD)		
CLBP (years)	17.3 (10.8)	12.6 (7.7)
CNP (years)	8.2 (11.1)	5.6 (6.8)
Diagnoses - # (%)		
CLBP	4 (29%)	3 (20%)
Undiagnosed CLBP	2 (14%)	2 (13%)
CLBP with undiagnosed CNP	1 (7%)	2 (13%)
Both	7 (50%)	8 (53%)

### Protocol Changes due to COVID-19

Changes to the study protocol were required for safety concerns during the COVID-19 pandemic. Thus, the project was delayed while planning for increased virtual research activities and more limited in-person activities. Placing research staff within various clinics had been our most productive modality for recruitment, but this was no longer possible. Recruitment was changed to online postings and health care provider referral only.

Screening was changed to hybrid virtual/in-person format and additional screening for the presence of COVID-related symptoms was required. Extra precautions were taken when bringing participants in for formal screening such as masking, distancing, and sanitizing rooms and equipment between participants. The additional COVID-related research requirements made screening and assessments more challenging and time-consuming, precluding group assessments or the ability to schedule multiple participants at the same time.

Interventions were initially scheduled to be held indoors at VA facilities. Masking became required in all areas of the Medical Center. Thus, we initially arranged for the Yoga + MR intervention to be held on a covered outdoor patio area at the medical facility to allow masks to be removed to encourage the deep breathing aspect of yoga. Because of construction occurring around the hospital this option also became infeasible and the remaining intervention sessions were held indoor with masks required.

#### Recruitment

**Figure 1.**
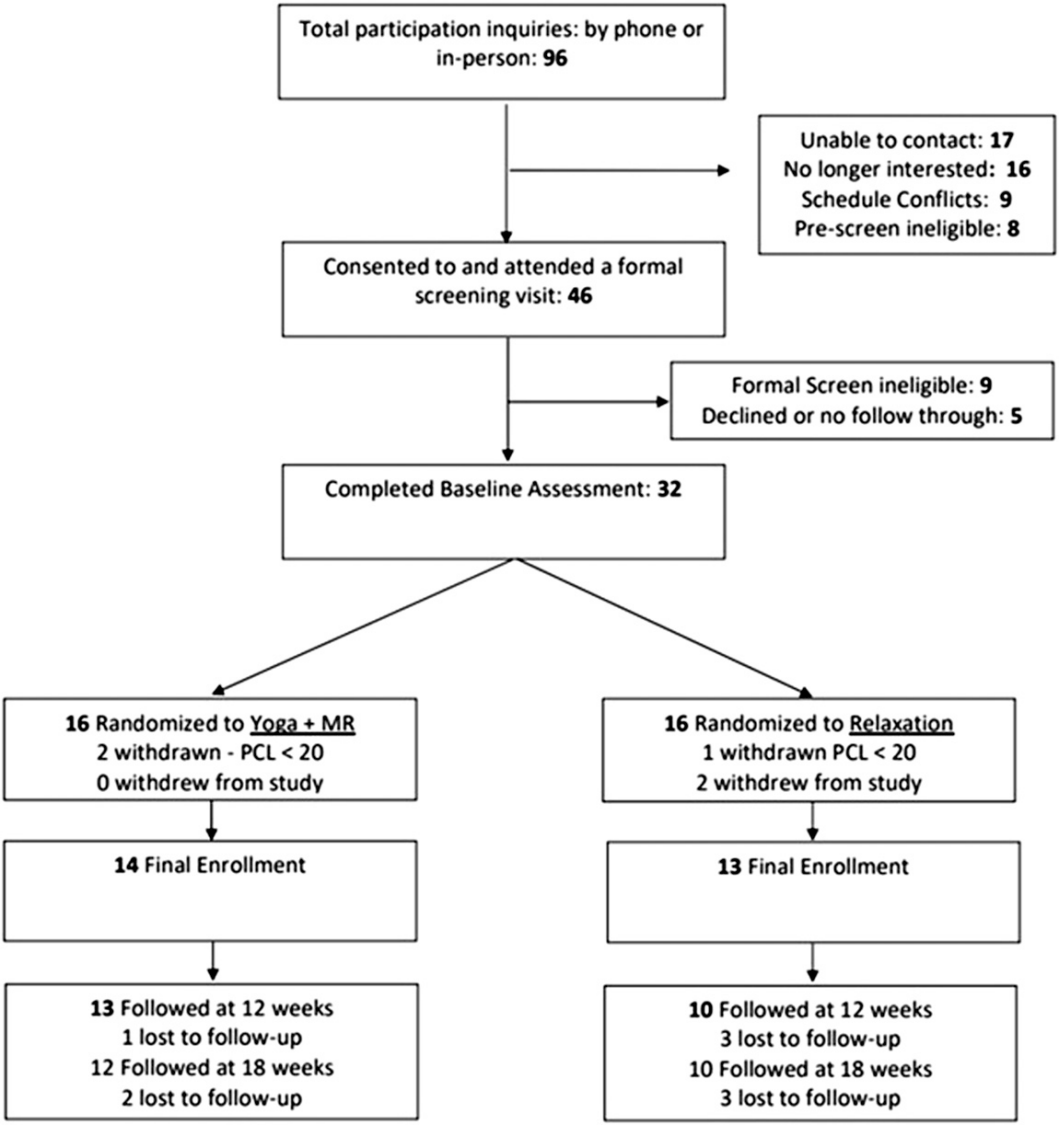
Participant flowchart.

The study recruited a total of 32 participants across 2 cohorts (cohort 1 = 17; cohort 2 = 15) over 11 months (See Figure 1) During this time, there were 96 inquiries or referrals of potential participants, of which 79 were successfully contacted by study staff. The leading source of referrals were from clinician referral from mental health (34) and pain (28) clinics. Of the 79 that were pre-screened by phone, 46 qualified to participate in the formal screen in person, 16 were no longer interested, eight were ineligible at the pre-screen, and nine were unavailable due to their schedule. After the formal screen, nine did not meet study criteria, and five participants qualified but declined to follow through by completing the baseline assessment. Therefore, 32 participants completed the informed consent process and the baseline assessment and were initially randomized.

#### Retention

Of the 32 participants initially randomized, three participants were unenrolled because their PCL-5 score was <20 and a current PTSD diagnosis could not be confirmed. Thus, they were ineligible. From an intent-to-treat perspective, 29 participants were eligible, leading to retention rates of 79% at the 12-week post-intervention follow-up, and 76% at the 18-week follow-up, 2 participants asked to be officially dropped from the study because they no longer wanted to participate. Among the 27 participants in the revised sample, retention rates were 85% at the 12-week post-intervention follow-up, and 79% at the 18-week follow-up, comfortably exceeding the minimum 75% retention goal at 12 weeks.

#### Attendance

Among all eligible participants, from an intent-to-treat perspective, mean attendance was 6.9 of 12 sessions or 58% overall. Attendance was not normally distributed, mostly because of six participants who attended zero or one session. By intervention group, mean attendance was 7.9 sessions or 66% for yoga + MR and 5.9 sessions or 49% for relaxation sessions. When excluding the two participants who were unable to attend and asked to be completely withdrawn from the study, mean attendance was 7.3 sessions or 61% overall (7.9 sessions or 66% for yoga + MR and 6.6 sessions or 55% for relaxation sessions). When excluding individuals who never attended an intervention session, mean attendance was 68% of the 12 sessions overall.

Participants were also encouraged to practice their skills at home and report days spent doing so each week. Participants in the relaxation intervention reported mean (SD) home practice of relaxation of 2.6 (1.1) days a week. Participants in the Yoga + MR class reported a mean (SD) of 2.8 (1.0) days/week of yoga home practice and a mean (SD) of 4.7 (1.7) days/week of MR home practice.

#### Adverse Events

No serious adverse events were reported for participants of either intervention. Other non-serious adverse events reported by participants were not found to be related to the interventions. Other adverse events recorded were non-COVID-related illnesses, COVID-19 exposures outside of the interventions, ongoing back or neck soreness, pain or PTSD symptoms related to activities outside of class. No COVID-19 infections were reported during the intervention period.

#### Intervention Satisfaction

Satisfaction with various aspects of the intervention was collected at the 12-week assessment (Table 2). The variables were negatively skewed with a high proportion of participants reporting high satisfaction. Satisfaction ratings were slightly higher among the Yoga + MR participants, with 100% agreeing or strongly agreeing that they enjoyed participation and liked the instructor. All but one Yoga + MR participant thought there were health benefits and one participant strongly disagreed that there were health benefits. That participant attended regularly for a while but reported increased pain and reduced function. Among the relaxation intervention participants, 1-2 participants were “neutral” on each dimension being rated.

**Table 2.** Program Satisfaction Ratings.

Program Evaluation	Yoga + MR (mean (SD); n = 13)	Relaxation (mean (SD); n = 8)	% Agree or Strongly Agree	% Disagree or Strongly Disagree
Enjoyed participating	4.62 (.51)	4.38 (.92)	100% - Y + MR 75% - relaxation	.0%
Experienced health benefits	4.15 (1.07)	4.38 (.74)	92% - Y + MR 88% - relaxation	4.3% (1 in YMR)
Enjoyed the instructor	4.77 (.44)	4.63 (.74)	100% - Y + MR 88% - relaxation	.0%

Likert Scale: Strongly Agree = 5; Agree = 4; Neutral = 3; Disagree = 2; Strongly Disagree = 1.

Qualitatively, feedback about both interventions was consistently positive. Relaxation group participants were younger on average, were more often women, and reported more attendance challenges because of work, school, and other obligations or commitments. We also received multiple unsolicited comments from participants across both groups expressing gratitude for the chance to attend in-person interventions. Many reported feeling stuck at home and isolated as a result of the COVID-19 pandemic.

#### Health Outcomes

Table 3 presents the mean scores on health outcomes at baseline and at 12 weeks (end of intervention). All variables met the assumption of distribution normality, with the exception of the AUDIT scores, for which a significant proportion of participants report no alcohol use. Change scores (with 95% confidence intervals) and pre-post effect sizes are also presented. Given the small sample sizes in this feasibility RCT, significance tests and between-group comparisons were not performed.^54^ Improvements were seen in both groups, but were inconsistent across outcomes. The Yoga + MR group had improvements including reduced back pain-related disability (primary outcome), reduced alcohol use, reduced fatigue, and increased quality of life on the visual analog scale. Among those assessed at 12 weeks in the relaxation group, reductions were observed in pain severity, PTSD symptoms, and fatigue. Means at 18 weeks are presented in the Supplementary Table.

**Table 3.** Health Outcomes by Group at Baseline and End-Of-Intervention (Means (SD); Pre-post Mean Difference (95% CI); Effect Size).

Outcome	Yoga + MR (n = 13)			Relaxation (n = 10)			Pre-post Cohen’s d	
	(Week 0)	(Week 12)	Mean difference (95% CI)	(Week 0)	(Week 12)	Mean difference (95% CI)	YMR	RC
Disability - RMDQ	13.5 (4.8)	9.8 (5.2)	−3.7 (−.1; −7.3)	14.5 (6.8)	13.2 (6.8)	−1.3 (−4.3; 1.7)	.62	.31
BPI – Pain severity	4.7 (1.9)	4.6 (2.3)	−.15 (.9; −1.2)	5.6 (1.9)	4.4 (2.4)	−1.2 (−2.4; .1)	.09	.64
BPI – Pain interference	5.2 (2.4)	4.5 (2.4)	−.73 (−2.1; .7)	6.2 (2.6)	5.7 (3.0)	−.5 (−2.1; 1.1)	.32	.20
PTSD - PCL-5	40.8 (15.2)	36.5 (19.5)	−4.2 (4.1; −12.6)	47.2 (17.7)	37.4 (21.8)	−9.8 (−18.8; −.8)	.31	.78
Insomnia - ISI	17.5 (6.0)	14.8 (6.3)	−2.7 (1.6; −7.0)	16.2 7.3)	15.9 (6.8)	−.3 (−3.1; 2.5)	.37	.08
Alcohol use - AUDIT-C	1.7 (2.4)	1.1 (1.9)	−.6 (−.1; −1.1)	1.4 (2.1)	1.3 (1.8)	−.1 (−.5; .3)	.71	.18
QOL - EQ5D	.61 (.19)	.64 (.17)	.03 (−.11; .16)	.48 (.20)	.58 (.31)	.11 (−.10; .31)	.11	.37
QOL - EQ5D VAS	56.8 (16.6)	64.4 (13.4)	7.6 (.9; 14.3)	63.9 (21.5)	64.4 (14.2)	.6 (−9.7, 10.8)	.69	.04
Fatigue – FSS	46.6 (10.8)	39.2 (10.6)	−7.4 (−1.7; −13.1)	41.5 (15.6)	36.6 (16.0)	−4.9 (−10.7; .9)	.79	.60
Depression - PHQ-9	12.2 (5.4)	10.6 (8.0)	−1.5 (.9; −4.0)	11.7 (6.9)	11.3 (8.2)	−.4 (−3.4; 2.6)	.39	.09

RMDQ, Roland-Morris Disability Questionnaire; NDI, Neck Disability Index; BPI-PS, Brief Pain Inventory-Pain Severity; BPI-PI, Brief Pain Inventory-Pain Interference; PROMIS-PI, PROMIS-Pain Intensity; SF-12-PH, Short-form Health Survey- Physical Health; SF-12-MH, Short-form Health Survey-Mental Health; FSS, Fatigue Severity Scale.

## Discussion

Despite challenges posed by the COVID-19 pandemic, the results of our feasibility study demonstrate support for a larger, fully-powered randomized clinical trial to study the benefits of Yoga + MR for cLBP/cNP and symptoms of PTSD. Recruitment and assessment activities were modified because of pandemic restrictions, enabling us to recruit and assess the target sample as planned. Attendance was good for the Yoga + MR intervention but was barely adequate for the relaxation intervention. Participant satisfaction with both interventions was high, and there were no safety concerns, including no serious adverse events reported. Finally, the outcomes suggest potential benefit that should be further explored in a larger study designed to examine effectiveness. Given the small sample sizes, lack of a normal distribution for some variables, and our focus on feasibility, the current study refrains from interpreting means and group differences beyond the recommended use of data from feasibility studies.^54,55^

A strength of the study was that the sample characteristics reflected racial and ethnic diversity, with 41% reporting Hispanic ethnicity and over 50% of those responding identifying as non-White race. (See Table 1). Fifteen percent of participants self-identified as women, above national rates indicating that 10% of US Veterans are women.^56^ About 60% of the sample reported military combat experience. PCL-5 scores at baseline were means of 41 in the Yoga + MR group and 47 in the Relaxation group, well above the cutoff of 31-33 on the PCL-5 that is indicative of PTSD.^57^

Recruitment was slowed by extra precautions during the pandemic that did not allow for in-person recruiting activities in VA clinics by study staff. Based on experience from prior RCTs conducted in the VA setting, we initially planned to recruit in-person, placing research staff in VA clinics so that VA health care providers could provide a “warm hand-off” to research staff for more information when patients were interested. Without this option during the pandemic, study staff regularly promoted and facilitated recruitment by providers by attending clinic provider meetings online, answering their questions, and reminding them about the study. Although this was the source of most referrals, we learned that a consequence of this approach was that some providers framed the study as a “yoga study”, creating expectations among some participants that they would receive yoga and not the relaxation control. Although preferences or expectations often emerge in many randomized trials, this issue can be minimized now that pandemic restrictions on research recruitment have returned to pre-pandemic levels. Other successful recruitment methods were advertisements through video screens in clinic waiting rooms and fliers posted throughout the medical facilities. Excluding the two participants who formally dropped out of the study, retention was 85% and 81% at the 12-week and 18-week follow-ups respectively, exceeding the 75% minimum retention goal. Participants appreciated the ability to complete their 12-week follow-up immediately after the last class to reduce the number of trips made to the hospital. Participants who did not complete follow-up assessments mainly consisted of those who did not attend class regularly or at all. Attempts to contact these participants to determine reasons for not responding had mixed success. Through phone calls and email, there were some indications that 1-2 participants were dissatisfied with their group assignment, while others cited scheduling conflicts and/or increased stress due to the pandemic and social or political events occurring during the 2020-2021 timeframe. One additional time-limited challenge during the study involved on-site construction at VASD. This made it harder for participants to get to assessments and intervention sessions at times.

Attendance of the Yoga + MR intervention (mean sessions attended = 66%) was consistent with rates from prior yoga intervention studies with veterans and/or military personnel.^23,58^ Excluding one participant that never attended yoga + MR, the mean attendance rate was 71%. Only one other participant attended less than half the sessions (5 sessions) and the median number of sessions attended was 9. This was augmented by home practice of both yoga (2.8 days/week) and the MR component (4.7 days/week), which appear to be substantial and near the range of home practice reported in a larger trial of lower-income non-veterans.^59^ Thus, solid home practice may offset less than optimal attendance in this study. In planning a larger follow-up study, efforts will focus on offering an additional Yoga + MR class, likely on weekends. We are also planning to expand recruitment and class offerings at a satellite clinic located in Oceanside, CA where yoga is currently being offered as part of the VA Whole Health program.

Attendance for the relaxation control intervention was lower and was just below the feasibility cutoff when viewed from the intent-to-treat perspective after two participants requested complete withdrawal from the study because of sudden conflicts with their work. Among those who remained in the study, attendance was at 55% but exceeded the feasibility cutoff of at least 50% of sessions. In-person relaxation classes were also augmented by home practice with participants reporting a mean of 3.0 days of relaxation exercise home practice. When comparing the two intervention groups, participants in the relaxation control were younger than those in the yoga intervention. Younger participants in the relaxation arm often cited conflicts in work schedules and family responsibilities as reasons for missing class. Future considerations for improving attendance include holding interventions at community locations which improve access for some Veterans. In addition, pandemic restrictions have been lifted for some time and seem unlikely to return at previous levels which will allow for in-person recruitment, no masking restrictions, and fewer concerns about being virally infected. Despite these expected differences in a larger study, the relaxation intervention will be re-evaluated for inclusion in a future study as discussed below.

To address intervention safety in this study, the study had to consider possible exposures to COVID-19 in addition to adverse events that could be associated with practicing yoga (most commonly increased pain). Participants were instructed to not attend intervention sessions or assessments and encouraged to get tested if they experienced any COVID-19 symptoms. In the Yoga + MR intervention, instructors were experienced, but received additional training to promote safe movements and modifications based on individual participant needs. Blankets, straps, and blocks were available to facilitate yoga positions. Home practice was strongly encouraged using a take-home manual with safely-designed activities. No major adverse events were reported indicating the safety of the program for future use. Illness was a common reason for missing class because of additional screening for symptoms of COVID-19, though illness or exposure were not attributed to intervention sessions.

Satisfaction was high in both groups, 92%–100% for the Yoga + MR group and 75%–88% for the relaxation control. In open-ended response sections, participants mentioned that they liked the MR component, and noted they were able to use MR in everyday settings to manage PTSD symptoms. One participant did not agree that there were health benefits and appeared to experience an increase in pain symptoms related to their neck. People with chronic pain have different pain etiologies and there is evidence that some poses may result in increased pain for a small proportion of participants.^60^ Most relaxation participants were similarly satisfied with their experience and with the instructor. One difference was that more relaxation participants expressed a desire for virtual class options. Reasons for this included reduced travel and not having to miss class for injury or illness. Although one relaxation participant indicated that symptoms of PTSD often made it hard to attend class, and that attending virtually from home would have been easier, many participants in both groups reported that they greatly appreciated having in-person instruction during the pandemic because of the individualized attention from the instructor and a desire to get out of their home more often.

Even though the study occurred during the COVID-19 pandemic amid contextual factors, some comparison of the feasibility findings to prior studies seems warranted. The target population for this study was VA patients with chronic pain and PTSD and both interventions were mind-body interventions. Among military veterans with PTSD, trauma-focused therapies have typically had low rates of treatment attendance and completion,^61,62^ but some brief exposure therapies have done better.^63^ For studies of mind-body interventions, a previous study of MR for PTSD among VA patients found trends toward greater non-completion of treatment in the MR group compared to present-centered therapy (22% vs 14%, respectively), but attendance was high among completers.^38^ However, this study involved individual therapy sessions only and had no focus on chronic pain. For yoga studies, a larger trial of yoga for PTSD in VA patients found that only 50% (54/108) of those assigned to yoga completed at least 8 or 16 sessions. In studies of yoga for chronic pain in veterans, two different studies found adherence or attendance of yoga to be less than optimal in this population,^23,64^ and lower than attendance in non-veteran community studies.^47,65^ Thus, despite being conducted during the pandemic, our feasibility results are similar to those found in studies of group-based mind-body interventions for veterans with chronic pain or PTSD.^23,28,64^

Health outcomes reflected sizable improvements in both intervention groups on some variables, but tests of significance and conclusions about efficacy are not appropriate given small sample sizes and our study design. Clinically important effects were obtained or approached by the mean scores of the Yoga + MR group for reductions in disability (primary outcome), fatigue, and alcohol use, along with increased quality of life.^66^ The relaxation group had important reductions in mean score on pain severity, PTSD symptoms, and fatigue.^66^ The improvements in the relaxation group suggest that the intervention could be efficacious in this population and is likely not an appropriate control condition for a superiority study design.

However, there were other factors in addition to the small sample size that make comparisons unwarranted with this data. For example, Yoga + MR participants were significantly older than relaxation group participants and there were no women in the Yoga + MR group. Although not statistically significant, Yoga + MR participants tended to be more educated and were less likely to report being disabled or unemployed. For baseline clinical characteristics, Yoga + MR participants tended to have lower scores on disability, pain, and PTSD symptoms, yet the duration of their chronic pain was longer. Thus, Yoga + MR participants appeared to have less severe, but more chronic, symptoms that could be more resistant to change. No systematic reason such as protocol non-adherence was found for these imbalances which likely occurred by chance given the small sample size. Thus, a larger study is needed to better balance these variables through randomization and be powered to detect minimally important differences prior to any firmer conclusions.

The impact and lessons learned from this feasibility study may be limited by the timing of the COVID-19 pandemic. It remains a strength that almost all study goals were accomplished despite pandemic restrictions, yet we cannot determine if similar or different results would occur otherwise. Adjustments because of the pandemic also led us to take a more flexible approach to modifying the intervention as needed to accommodate pandemic restrictions. Thus, we did not formally assess intervention fidelity which may be seen as a limitation. In the end, neither the Yoga + MR nor relaxation intervention were notably altered, but masking requirements led to challenges with intervention breathwork and some sessions did not complete every pose listed within the allocated time.

Although increased pain was reported by one individual in the Yoga + MR group, the instructors did not receive feedback that specific yoga poses (asanas) were linked to this increase. Small increases in pain for some participants were expected early in the intervention, and instructors encouraged participants to not push themselves, to communicate about discomfort, and to seek modifications. The individual reporting increased pain entered the study with both cLBP and CNP which were a vulnerability in a prior study,^58^ in addition to active PTSD. We conclude that extra attention should be paid when working with military veterans who may be unaccustomed to admitting increased pain, especially in a group setting.^67^

In conclusion, our data suggest the interventions were acceptable and safe, and that the feasibility of conducting a larger RCT was established. Feasibility was established despite the study being conducted mainly in 2021 with numerous challenges related to the COVID-19 pandemic. The study staff and participants may also have been personally affected by the pandemic and by social and political events that were present during the study. Although many studies shifted to remotely delivered interventions during the pandemic, transitioning the study to remote delivery posed its own practical and potential challenges. Thus, we decided on in-person interventions and it was notable that many participants expressed gratitude for in-person activities due to the ongoing pandemic, feelings of isolation, and a desire to interact with others in-person. Based on our results, a larger study is being planned including changes to the study design and choice of comparison group (or groups). Options include comparing yoga + MR to a health education control or moving toward a comparative effectiveness design with pain-related function the primary outcome.

## Supplemental Material

Supplemental Material - Yoga Plus Mantram Repetition to Reduce Chronic Pain in Veterans With Post-Traumatic Stress Disorder: A Feasibility Trial Click here for additional data file. Supplemental Material for Yoga Plus Mantram Repetition to Reduce Chronic Pain in Veterans With Post-Traumatic Stress Disorder: A Feasibility Trial by Erik J. Groessl, Carol L. Hafey, Adhana McCarthy, Rahil M. Hernandez, Miguel Prado-Nava, Danielle Casteel, Symone McKinnon, Douglas G. Chang, Catherine R. Ayers, Thomas R. Rutledge, Ariel J. Lang, and Jill E. Bormann in Global Advances in Integrative Medicine and Health

## References

[1] Coleman BC Goulet JL Higgins DM , et al. ICD-10 coding of musculoskeletal conditions in the veterans health administration. Pain Med. 2021;22(11):2597-2603. 33944953 10.1093/pm/pnab161 PMC8783617

[2] Goulet JL Kerns RD Bair M , et al. The musculoskeletal diagnosis cohort: examining pain and pain care among veterans. Pain. 2016;157(8):1696-1703. 27023420 10.1097/j.pain.0000000000000567 PMC4949131

[3] Roy TC . Diagnoses and mechanisms of musculoskeletal injuries in an infantry brigade combat team deployed to Afghanistan evaluated by the brigade physical therapist. Mil Med. 2011;176(8):903-908. 21882780 10.7205/milmed-d-11-00006

[4] Patzkowski JC Rivera JC Ficke JR Wenke JC . The changing face of disability in the US army: the operation enduring freedom and operation Iraqi freedom effect. J Am Acad Orthop Surg. 2012;20(Suppl 1):S23-30. 22865131 10.5435/JAAOS-20-08-S23

[5] Chou R Qaseem A Snow V , et al. Diagnosis and treatment of low back pain: a joint clinical practice guideline from the American College of Physicians and the American Pain Society. Ann Intern Med. 2007;147(7):478-491. 17909209 10.7326/0003-4819-147-7-200710020-00006

[6] Gore M Tai KS Sadosky A Leslie D Stacey BR . Use and costs of prescription medications and alternative treatments in patients with osteoarthritis and chronic low back pain in community-based settings. Pain Pract. 2012;12(7):550-560. 22304678 10.1111/j.1533-2500.2012.00532.x

[7] Bohnert AS Ilgen MA Trafton JA , et al. Trends and regional variation in opioid overdose mortality among Veterans Health Administration patients, fiscal year 2001 to 2009. Clin J Pain. 2014;30(7):605-612. 24281278 10.1097/AJP.0000000000000011

[8] Ducoffe AR York A Hu DJ Perfetto D Kerns RD . National action plan for adverse drug event prevention: recommendations for safer outpatient opioid use. Pain Med. 2016;17:2291-2304. 28025363 10.1093/pm/pnw106 PMC6280931

[9] Dorflinger L Moore B Goulet J , et al. A partnered approach to opioid management, guideline concordant care and the stepped care model of pain management. J Gen Intern Med. 2014;29(Suppl 4):870-876. 10.1007/s11606-014-3019-2 PMC4239281 25355083

[10] Pangarkar SS Kang DG Sandbrink F , et al. VA/DoD clinical practice guideline: diagnosis and treatment of low back pain. J Gen Intern Med. 2019;34(11):2620-2629. 31529375 10.1007/s11606-019-05086-4 PMC6848394

[11] Qaseem A McLean RM O'Gurek D Batur P Lin K Kansagara DL . Nonpharmacologic and pharmacologic management of acute pain from non-low back, musculoskeletal injuries in adults: a clinical guideline from the American college of physicians and American academy of family physicians. Ann Intern Med. 2020;173(9):739-748. 32805126 10.7326/M19-3602

[12] Byers AL Covinsky KE Neylan TC Yaffe K . Chronicity of posttraumatic stress disorder and risk of disability in older persons. JAMA Psychiatr. 2014;71(5):540-546. 10.1001/jamapsychiatry.2014.5 PMC4119004 24647756

[13] Mendlowicz MV Stein MB . Quality of life in individuals with anxiety disorders. Am J Psychiatr. 2000;157(5):669-682. 10784456 10.1176/appi.ajp.157.5.669

[14] Moeller-Bertram T Afari N Mostoufi S Fink DS Johnson Wright L Baker DG . Specific pain complaints in Iraq and Afghanistan veterans screening positive for post-traumatic stress disorder. Psychosomatics. 2014;55(2):172-178. 23473449 10.1016/j.psym.2013.01.011

[15] Outcalt SD Ang DC Wu J Sargent C Yu Z Bair MJ . Pain experience of Iraq and Afghanistan Veterans with comorbid chronic pain and posttraumatic stress. J Rehabil Res Dev. 2014;51(4):559-570. 25144169 10.1682/JRRD.2013.06.0134

[16] Benedict TM Keenan PG Nitz AJ Moeller-Bertram T . Post-traumatic stress disorder symptoms contribute to worse pain and health outcomes in veterans with PTSD compared to those without: a systematic review with meta-analysis. Mil Med. 2020;185(9-10):e1481-e1491. 32248229 10.1093/milmed/usaa052

[17] Gros DF Szafranski DD Brady KT Back SE . Relations between pain, PTSD symptoms, and substance use in veterans. Psychiatry. 2015;78(3):277-287. 26391835 10.1080/00332747.2015.1069659 PMC4867497

[18] Seal KH Shi Y Cohen G , et al. Association of mental health disorders with prescription opioids and high-risk opioid use in US veterans of Iraq and Afghanistan. JAMA. 2012;307(9):940-947. 22396516 10.1001/jama.2012.234

[19] Cifu DX Taylor BC Carne WF , et al. Traumatic brain injury, posttraumatic stress disorder, and pain diagnoses in OIF/OEF/OND Veterans. J Rehabil Res Dev. 2013;50(9):1169-1176. 24458958 10.1682/JRRD.2013.01.0006

[20] Stojanovic MP Fonda J Fortier CB , et al. Influence of mild traumatic brain injury (TBI) and posttraumatic stress disorder (PTSD) on pain intensity levels in OEF/OIF/OND veterans. Pain Med. 2016;17(11):2017-2025. 27040665 10.1093/pm/pnw042

[21] Chou R Deyo R Friedly J , et al. Nonpharmacologic therapies for low back pain: a systematic review for an American college of physicians clinical practice guideline. Ann Intern Med. 2017;166(7):493-505. 28192793 10.7326/M16-2459

[22] Goldstein E McDonnell C Atchley R , et al. The impact of psychological interventions on posttraumatic stress disorder and pain symptoms: a systematic review and meta-analysis. Clin J Pain. 2019;35(8):703-712. 31145146 10.1097/AJP.0000000000000730

[23] Groessl EJ Liu L Chang DG , et al. Yoga for military veterans with chronic low back pain: a randomized clinical trial. Am J Prev Med. 2017;53(5):599-608. 28735778 10.1016/j.amepre.2017.05.019 PMC6399016

[24] Goode AP Coeytaux RR McDuffie J , et al. An evidence map of yoga for low back pain. Compl Ther Med. 2016;25:170-177. 10.1016/j.ctim.2016.02.016 27062965

[25] Wieland LS Skoetz N Pilkington K Vempati R D'Adamo CR Berman BM . Yoga treatment for chronic non-specific low back pain. Cochrane Database Syst Rev. 2017;1:CD010671. 28076926 10.1002/14651858.CD010671.pub2 PMC5294833

[26] Li Y Li S Jiang J Yuan S . Effects of yoga on patients with chronic nonspecific neck pain: a PRISMA systematic review and meta-analysis. Medicine. 2019;98(8):e14649. 30813206 10.1097/MD.0000000000014649 PMC6407933

[27] Cramer H Anheyer D Saha FJ Dobos G . Yoga for posttraumatic stress disorder - a systematic review and meta-analysis. BMC Psychiatr. 2018;18(1):72. 10.1186/s12888-018-1650-x PMC5863799 29566652

[28] Davis LW Schmid AA Daggy JK , et al. Symptoms improve after a yoga program designed for PTSD in a randomized controlled trial with veterans and civilians. Psychol Trauma. 2020;12(8):904-912. 32309986 10.1037/tra0000564

[29] Beck D Cosco Holt L Burkard J , et al. Efficacy of the mantram repetition program for Insomnia in veterans with posttraumatic stress disorder: a naturalistic study. ANS Adv Nurs Sci. 2016;40:E1-E12. 10.1097/ANS.0000000000000144 27525960

[30] Bormann JE Oman D Walter KH Johnson BD . Mindful attention increases and mediates psychological outcomes following mantram repetition practice in veterans with posttraumatic stress disorder. Med Care. 2014;52(12 Suppl 5):S13-18. 25397817 10.1097/MLR.0000000000000200

[31] Bormann JE Thorp SR Wetherell JL Golshan S Lang AJ . Meditation-based mantram intervention for veterans with posttraumatic stress disorder: a randomized trial. Psychological Trauma: Theory, Research, Practice and Policy. 2013;5(3):259-267.

[32] Hilton L Maher AR Colaiaco B , et al. Meditation for posttraumatic stress: systematic review and meta-analysis. Psychol Trauma. 2016;9:453-460. 27537781 10.1037/tra0000180

[33] Lang AJ Strauss JL Bomyea J , et al. The theoretical and empirical basis for meditation as an intervention for PTSD. Behav Modif. 2012;36(6):759-786. 22669968 10.1177/0145445512441200

[34] Lacefield K Samph SP Orbon S Otis J . Integrated intervention for comorbid posttraumatic stress disorder and fibromyalgia: a pilot study of women veterans. Psychol Trauma. 2020;12(7):725-729. 32757579 10.1037/tra0000635

[35] Otis JD Keane TM Kerns RD Monson C Scioli E . The development of an integrated treatment for veterans with comorbid chronic pain and posttraumatic stress disorder. Pain Med. 2009;10(7):1300-1311. 19818040 10.1111/j.1526-4637.2009.00715.x

[36] Plagge JM Lu MW Lovejoy TI Karl AI Dobscha SK . Treatment of comorbid pain and PTSD in returning veterans: a collaborative approach utilizing behavioral activation. Pain Med. 2013;14(8):1164-1172. 23746043 10.1111/pme.12155

[37] Chopin SM Sheerin CM Meyer BL . Yoga for warriors: an intervention for veterans with comorbid chronic pain and PTSD. Psychol Trauma. 2020;12(8):888-896. 32700935 10.1037/tra0000649 PMC7909482

[38] Bormann JE Thorp SR Smith E , et al. Individual treatment of posttraumatic stress disorder using mantram repetition: a randomized clinical trial. Am J Psychiatr. 2018;175(10):979-988. 29921143 10.1176/appi.ajp.2018.17060611

[39] VA Rehabilitation Research and Development Service (RR&D) . RR&D Mission. https://www.rehab.research.va.gov/. Published 2023. Accessed 10/06/23.

[40] Emerson D Sharma R Chaudhry S Turner J . Trauma-sensitive yoga: principles, practice and research. International Journal of Yoga Therapy. 2009;19:123-128.

[41] Groessl EJ Schmalzl L Maiya M , et al. Yoga for veterans with chronic low back pain: design and methods of a randomized clinical trial. Contemp Clin Trials. 2016;48:110-118. 27103548 10.1016/j.cct.2016.04.006

[42] Iyengar BKS . Light on Yoga. Revised ed. New York, NY: Schocken; 1979.

[43] Lang AJ Malaktaris AL Casmar P , et al. Compassion meditation for posttraumatic stress disorder in veterans: a randomized proof of concept study. J Trauma Stress. 2019;32(2):299-309. 30929283 10.1002/jts.22397

[44] Taylor S Thordarson DS Maxfield L Fedoroff IC Lovell K Ogrodniczuk J . Comparative efficacy, speed, and adverse effects of three PTSD treatments: exposure therapy, EMDR, and relaxation training. J Consult Clin Psychol. 2003;71(2):330-338. 12699027 10.1037/0022-006x.71.2.330

[45] Saper RB Boah AR Keosaian J Cerrada C Weinberg J Sherman KJ . Comparing once- versus twice-weekly yoga classes for chronic low back pain in predominantly low income minorities: a randomized dosing trial. Evid Based Complement Alternat Med. 2013;2013:658030. 23878604 10.1155/2013/658030 PMC3710634

[46] Sherman KJ Cherkin DC Wellman RD , et al. A randomized trial comparing yoga, stretching, and a self-care book for chronic low back pain. Arch Intern Med. 2011;171:2019-2026. 22025101 10.1001/archinternmed.2011.524 PMC3279296

[47] Tilbrook HE Cox H Hewitt CE , et al. Yoga for chronic low back pain: a randomized trial. Ann Intern Med. 2011;155(9):569-578. 22041945 10.7326/0003-4819-155-9-201111010-00003

[48] Furlan AD Pennick V Bombardier C van Tulder M Editorial Board Cochrane Back Review Group . 2009 updated method guidelines for systematic reviews in the Cochrane Back Review Group. Spine. 2009;34(18):1929-1941. 19680101 10.1097/BRS.0b013e3181b1c99f

[49] Sherman KJ Cherkin DC Cook AJ , et al. Comparison of yoga versus stretching for chronic low back pain: protocol for the Yoga Exercise Self-care (YES) trial. Trials. 2010;11:36. 20356395 10.1186/1745-6215-11-36 PMC2864260

[50] Hohenschurz-Schmidt D Kleykamp BA Draper-Rodi J , et al. Pragmatic trials of pain therapies: a systematic review of methods. Pain. 2022;163(1):21-46. 34490854 10.1097/j.pain.0000000000002317 PMC8675058

[51] Herdman M Gudex C Lloyd A , et al. Development and preliminary testing of the new five-level version of EQ-5D (EQ-5D-5L). Qual Life Res. 2011;20(10):1727-1736. 21479777 10.1007/s11136-011-9903-x PMC3220807

[52] Krupp LB LaRocca NG Muir-Nash J Steinberg AD . The fatigue severity scale. Application to patients with multiple sclerosis and systemic lupus erythematosus. Arch Neurol. 1989;46(10):1121-1123. 2803071 10.1001/archneur.1989.00520460115022

[53] Kroenke K Spitzer RL Williams JB . The PHQ-9: validity of a brief depression severity measure. J Gen Intern Med. 2001;16(9):606-613. 11556941 10.1046/j.1525-1497.2001.016009606.x PMC1495268

[54] Leon AC Davis LL Kraemer HC . The role and interpretation of pilot studies in clinical research. J Psychiatr Res. 2011;45(5):626-629. 21035130 10.1016/j.jpsychires.2010.10.008 PMC3081994

[55] Whitehead AL Sully BG Campbell MJ . Pilot and feasibility studies: is there a difference from each other and from a randomised controlled trial? Contemp Clin Trials. 2014;38(1):130-133. 24735841 10.1016/j.cct.2014.04.001

[56] Women Veterans Report: The Past, Present, and Future of Women Veterans. Washington, DC: National Center for Veterans Analysis and Statistics, Department of Veterans Affairs. https://www.va.gov/vetdata/docs/specialreports/women_veterans_2015_final.pdf (2017).

[57] Bovin MJ Marx BP Weathers FW , et al. Psychometric properties of the PTSD checklist for diagnostic and statistical manual of mental disorders-fifth edition (PCL-5) in veterans. Psychol Assess. 2016;28(11):1379-1391. 26653052 10.1037/pas0000254

[58] Groessl EJ Casteel D McKinnon S , et al. Comparing types of yoga for chronic low back and neck pain in military personnel: a feasibility randomized controlled trial. Glob Adv Health Med. 2022;11:2164957X221094596. 10.1177/2164957X221094596 PMC9208032 35734420

[59] Saper RB Lemaster C Delitto A , et al. Yoga, physical therapy, or education for chronic low back pain: a randomized noninferiority trial. Ann Intern Med. 2017;167(2):85-94. 28631003 10.7326/M16-2579 PMC6392183

[60] Campo M Shiyko MP Kean MB Roberts L Pappas E . Musculoskeletal pain associated with recreational yoga participation: a prospective cohort study with 1-year follow-up. J Bodyw Mov Ther. 2018;22(2):418-423. 29861244 10.1016/j.jbmt.2017.05.022

[61] Foy DW . Group therapies for trauma using cognitive-behavioral therapy. In: Follette VM Ruzek JI , ed. Cognitive-behavioral Therapies for Trauma. New York: Guilford Press; 2006:388-404.

[62] Hoge CW Terhakopian A Castro CA Messer SC Engel CC . Association of posttraumatic stress disorder with somatic symptoms, health care visits, and absenteeism among Iraq war veterans. Am J Psychiatr. 2007;164(1):150-153. 17202557 10.1176/ajp.2007.164.1.150

[63] Kip KE Rosenzweig L Hernandez DF , et al. Accelerated Resolution Therapy for treatment of pain secondary to symptoms of combat-related posttraumatic stress disorder. Eur J Psychotraumatol. 2014;5:24066. 10.3402/ejpt.v5.24066 PMC4014659 24959325

[64] Roseen EJ Pinheiro A Lemaster CM , et al. Yoga versus education for veterans with chronic low back pain: a randomized controlled trial. J Gen Intern Med. 2023;38(9):2113-2122. 36650329 10.1007/s11606-023-08037-2 PMC10361953

[65] Sherman KJ Cherkin DC Wellman RD , et al. A randomized trial comparing yoga, stretching, and a self-care book for chronic low back pain. Arch Intern Med. 2011;171(22):2019-2026. 22025101 10.1001/archinternmed.2011.524 PMC3279296

[66] Ostelo RW Deyo RA Stratford P , et al. Interpreting change scores for pain and functional status in low back pain: towards international consensus regarding minimal important change. Spine. 2008;33(1):90-94. 18165753 10.1097/BRS.0b013e31815e3a10

[67] Hurst S Maiya M Casteel D , et al. Yoga therapy for military personnel and veterans: qualitative perspectives of yoga students and instructors. Compl Ther Med. 2018;40:222-229. 10.1016/j.ctim.2017.10.008 PMC6935718 30219455

