# Supplementary material for: Yoga Plus Mantram Repetition to Reduce Chronic Pain in Veterans With Post-Traumatic Stress Disorder: A Feasibility Trial
Source: Glob Adv Integr Med Health. 2023 Dec 26;12:27536130231220623. doi: 10.1177/27536130231220623 (PMC10752061; doi:10.1177/27536130231220623)
Supplement: Supplemental Material - Yoga Plus Mantram Repetition to Reduce Chronic Pain in Veterans With Post-Traumatic Stress Disorder: A Feasibility Trial [file sj-pdf-1-gam-10.1177_27536130231220623.pdf]

Supplementary Table 1 – Health Outcomes by group at baseline, end-of-intervention, and 18-week follow-up (means (sd))

	Yoga+MR			Relaxation		
**Outcome**	(Week 0) n=13	(Week 12) n=13	(Week 18) n=12	(Week 0) n=10	(Week 12) n=10	(Week 18) n=10
Disability - RMDQ	13.5 (4.8)	9.8 (5.2)	10.0 (5.7)	14.5 (6.8)	13.2 (6.8)	15.6 (6.2)
BPI – Pain Severity	4.7 (1.9)	4.6 (2.3)	4.88 (1.4)	5.6 (1.9)	4.4 (2.4)	4.88 (1.9)
BPI – Pain Interference	5.2 (2.4)	4.5 (2.4)	5.5 (2.0)	6.2 (2.6)	5.7 (3.0)	5.1 (2.5)
PTSD - PCL-5	40.8 (15.2)	36.5 (19.5)	49.6 (10.9)	47.2 (17.7)	37.4 (21.8)	44.1 (22.2)
Insomnia - ISI	17.5 (6.0)	14.8 (6.3)	18.8 (5.2)	16.2 7.3)	15.9 (6.8)	16.9 (6.2)
Alcohol use - AUDIT-C	1.7 (2.4)	1.1 (1.9)	1.3 (2.6)	1.4 (2.1)	1.3 (1.8)	0.90 (1.0)
QOL - EQ5D	0.61 (0.19)	0.64 (0.17)	0.57 (0.18)	0.48 (0.20)	0.58 (0.31)	0.49 (0.28)
QOL - EQ5D VAS	56.8 (16.6)	64.4 (13.4)	57.5 (18.8)	63.9 (21.5)	64.4 (14.2)	56.4 (18.7)
Fatigue – FSS	46.6 (10.8)	39.2 (10.6)	43.7 (11.8)	41.5 (15.6)	36.6 (16.0)	40.1 (17.0)
Depression - PHQ-9	12.2 (5.4)	10.6 (8.0)	15.6 (4.8)	11.7 (6.9)	11.3 (8.2)	12.0 (8.3)

RMDQ=Roland-Morris Disability Questionnaire; NDI=Neck Disability Index; BPI-PS= Brief Pain Inventory-Pain Severity; BPI-PI= Brief Pain Inventory-Pain Interference; PROMIS-PI = PROMIS-Pain Intensity; SF-12-PH= Short-form Health Survey- Physical Health; SF-12-MH= Short-form Health Survey-Mental Health; FSS= Fatigue Severity Scale.

For Peer Review
